# Virtual nurse support to enhance antipsychotic adherence in schizophrenia: A South African perspective

**DOI:** 10.4102/sajpsychiatry.v31i0.2430

**Published:** 2025-05-31

**Authors:** Yasmeen Thandar, Nomhle Mvunelo, Deepak Singh, Firoza Haffejee

**Affiliations:** 1 Department of Basic Medical Sciences, Faculty of Health Sciences, Durban University of Technology, Durban, South Africa; 2 Department of Physics, Faculty of Applied Sciences, Durban University of Technology, Durban, South Africa

**Keywords:** schizophrenia, adherence, antipsychotics, psychiatry, treatment buddy, telemedicine, psychiatric nursing

## Abstract

**Background:**

Patients with schizophrenia rely on antipsychotic medication, with adherence being critical for symptom management. Poor adherence leads to relapse, disability and increased healthcare costs.

**Aim:**

This study investigated the impact on antipsychotic adherence among schizophrenia patients on the introduction of an intervention utilising a psychiatric nurse as a virtual treatment buddy.

**Setting:**

Participants were recruited from a psychiatric clinic in KwaZulu-Natal, South Africa.

**Methods:**

This quantitative pre-test-post-test control group study recruited 117 schizophrenia patients. Participants were randomly assigned to an intervention group (*n* = 82) receiving daily text message support or a control group (*n* = 35) without support. Pre- and post-intervention questionnaires assessed medication adherence. The McNemar test compared medication cessation frequencies pre- and post-intervention. Pearson’s chi-squared test, factor analysis and linear regressions were employed to correlate variables. A *p* < 0.05 was deemed statistically significant.

**Results:**

Adherence improved from 60.8% to 83.51% post-intervention (*p* < 0.001). Obstacles to adherence, including inadequate disease knowledge, treatment duration, side effects, misunderstanding instructions and forgetfulness, were reduced post-intervention. Additionally, symptom relapse, rehospitalisation, specialist referrals, the need for more potent medication and employment loss decreased post-intervention.

**Conclusion:**

Virtual buddy support has demonstrated promise in improving medication adherence and minimising adverse effects of discontinuation among schizophrenia patients.

**Contribution:**

This study contributes a method to improving antipsychotic adherence and reducing negative outcomes in schizophrenia patients by emphasising personalised support, education and collaborative care among healthcare providers and support systems.

## Introduction

Schizophrenia is a severe psychiatric disorder marked by impaired communication, loss of reality and a decline in work, social functioning and self-care.^1^ Unlike anxiety and depression, where treatment can restore normal functioning, schizophrenia is a chronic condition requiring lifelong management and causes lasting social and work impairment.^2^ Treatment incorporates antipsychotic medication with psychosocial approaches like cognitive behavioural therapy, psychoeducation, family intervention, motivational interviewing, social skills training and assertive community treatment.^3^

Adherence to medication has been defined as the extent to which patients take their prescribed medication.^4^ Healthcare professionals view treatment adherence as vital for recovery during acute and long-term management of mental illness.^5^ Recent reports indicate that around 56% of the schizophrenia patients are non-adherent to their antipsychotics.^6^ A South African study reported adherence to antipsychotics for only 2 months, causing relapse.^7^ Low adherence increases economic costs from relapses, re-hospitalisations, condition decline and productivity reduction.^8^ Causes of non-adherence include side effects, poverty, lack of family support, long illness duration, stigma, alcohol consumption and smoking.^9^ Challenges in managing chronic mental disorders include frequent daily dosing, multiple drugs, forgetfulness, reluctance for long-term continuation and high costs.^10^ Financial stability, family support, supervised medication intake by healthcare professionals or family members, once-daily medications with symptom relief, fewer side effects, understanding the condition and positive health beliefs are key factors that enhance adherence.^11^

Nurses play a positive role in changing patients’ attitudes and insights towards their disease and increasing medication adherence.^12^ A qualitative study on mental health nurses’ strategies for promoting medication adherence in schizophrenia identified key themes: establishing a conversational relationship, assessing non-adherence, understanding the disease, incorporating feedback and building supportive resources.^12^ Dearing (2004) emphasised that in the nurse-patient relationship, trust rooted in co-operation and support is essential for compliance.^13^

Fostering a patient--therapist therapeutic alliance, reminder systems and addressing substance use disorder have also been reported to improve adherence.^14,15^ Systematic review analyses have found that text messaging interventions enhance medication adherence and clinical outcomes in serious mental health disorders and substance use disorders.^16,17^ None of the included studies were based in Africa.

In Southern Africa, high patient volumes and staff shortages generate long waiting times at healthcare facilities, reducing patient satisfaction and discouraging attendance, which impacts adherence.^18,19,20^ A recent South African study on nurses’ experiences in the public sector highlighted challenges within the healthcare system. One major issue is the high patient load daily, which compromises the quality of care. Some reported resorting to ‘slap dashes’ to manage the queue.^21^ This reflects a lack of personalised treatment, potentially impacting adherence.

Treatment supporters have proven successful in various diseases, including human immunodeficiency virus/acquired immune deficiency syndrome (HIV/AIDS), tuberculosis through the directly observed treatment (DOT) programme and cancer.^22,23,24,25^ In South Africa, treatment partners and text messaging to improve adherence to psychotropic medication were previously explored in a qualitative study.^26^ While most patients endorsed treatment supporters, some were concerned about overly controlling partners and loss of autonomy.^26^

Peer supporters in mental healthcare have demonstrated positive influence in improving adherence and engagement and strengthening connections with healthcare providers.^27,28,29,30,31,32,33,34^ These individuals, often survivors of mental illness, can be peer companions, advocates or counsellors.^34^ Treatment buddies, however, are trusted family members or friends who support patients with chronic conditions. They may accompany patients to clinics, provide lay counselling and help establish healthy behaviours.^23^

This study utilised a psychiatric nurse as a virtual treatment buddy with an aim to enhance adherence to schizophrenia treatment and minimise consequences of non-adherence. Virtual support instead of physical presence was utilised because of clinic regulations to prevent overcrowding and viral transmission during the coronavirus disease 2019 (COVID-19) pandemic.

We investigated adherence levels, factors influencing adherence, effects of non-adherence and treatment buddy preferences among schizophrenia patients exposed to the virtual treatment buddy intervention through a self-reported feedback questionnaire.

## Research methods and design

### Research design

This quantitative study employed a quasi-experimental pre-test-post-test control group design. Participants were randomly assigned by the primary investigator to either an intervention group or a control group, with every third patient allocated to the control group. Outcomes were evaluated through questionnaire responses. Although not a randomised controlled trial, Consolidated Standards of Reporting Trials (CONSORT) guidelines were followed to ensure accurate reporting.^35^

The control group received standard care, which included monthly clinic visits for script renewal and medication collection, during which they were attended to by a psychiatric nurse. The intervention group received the same standard care as well as 6 months of virtual treatment buddy support from the primary investigator, a psychiatric nurse. Recruitment occurred over 3 weeks during the COVID-19 pandemic from June 2021 to July 2021, with data collection spanning from June 2021 to December 2021.

The primary role of the virtual treatment buddy was to deliver standardised daily text reminders via WhatsApp or short messaging service (SMS) to participants in the intervention group, ensuring medication adherence and timely clinic visits. However, as participants could respond to the messages, the role organically expanded beyond reminders to include responding to participant enquiries and providing support. Periodic random phone calls were also conducted to verify message receipt, and this encouraged engagement.

A key advantage was that the buddy was a psychiatric nurse, allowing for appropriate responses to treatment-related concerns and basic psychosocial support. This professional expertise enhanced the overall support provided through the virtual platform.

The control group received no treatment buddy support. Both groups completed a pre-intervention questionnaire to assess medication adherence before the study and a post-intervention questionnaire to evaluate adherence after 6 months of treatment buddy support.

Questionnaires were validated by a three-member expert panel, including the primary researcher, a psychiatric nurse, a public health research professor and a research pharmacologist. Each question was critically reviewed, ensuring alignment with the study objectives and clinical relevance, then refined and agreed upon before piloting, ensuring validity and reliability. Both English and isiZulu versions were available, as isiZulu is a predominant language in the study area. The pre- and post-intervention questionnaires included subsections on demographic data, treatment adherence, factors influencing non-adherence, effects of non-adherence and participants’ experiences and preferences regarding treatment buddies. Both groups completed the post-intervention questionnaire although some sections were not applicable to the control group.

### Study population

A non-probability, convenience sampling method was utilised to recruit participants attending the Chatsworth Psychiatric Clinic at RK (CPCRK) Hospital in KwaZulu-Natal (KZN), South Africa.

Inclusion criteria consisted of patients diagnosed with schizophrenia and prescribed psychiatric treatment for at least 6 months by a psychiatric healthcare practitioner in KZN. Participants also had to have been on a long-term treatment plan of at least 1 year, be on a monthly schedule of script renewal and collecting medication at the study site. Those below 18 years, over 65 years, and having an intellectual disability were excluded.

**FIGURE 1:**
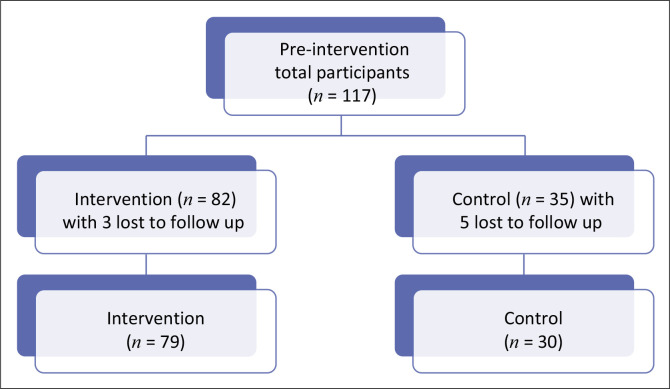
Total number of participants pre-intervention, intervention and control.

The study population consisted of approximately 220 patients with schizophrenia attending CPCRK. In collaboration with a statistician and utilising the Clincalc^®^ sample size calculator, with a 0.05 significance level and 80% power, the minimum sample sizes were calculated as 58 for the intervention group and 29 for the control group. To account for potential loss to follow-up, recruitment targets were set at 82 for the intervention group and 35 for the control group. There were 117 participants recruited, with 8 participants lost to follow-up (3 intervention and 5 control) because of death (1) and inability to be located (7). The final participants’ numbers in the intervention and control groups are reflected in Figure 1.

Participants were approached by the primary researcher, given a participant information letter, and asked to sign a consent form if interested. Questionnaires were completed in a private room, with the researcher available for questions. Pre-intervention data collection lasted 3 weeks based on participant availability. Both groups were contacted at the end of the 6-month study period to complete the post-intervention questionnaire.

### Data analysis

Data were analysed using a per-protocol approach, focusing on participants who fully adhered to the protocol to minimise non-adherence effects. The data were coded, captured in a spreadsheet and analysed using the Statistical Package for the Social Sciences (SPSS) (Version 25). Descriptive statistics, including frequencies, means and standard deviations, were calculated as appropriate. The McNemar test compared the proportions of stopping medications between pre- and post-intervention groups. Correlations among variables were assessed using Pearson’s chi-squared test, factor analysis and linear regression as appropriate, with a *p* < 0.05 considered statistically significant.

### Ethical considerations

The Institutional Research Ethics Committee (IREC) of the Durban University of Technology provided ethical clearance (IREC 030/20). Permission to conduct the study was granted by the KZN Department of Health and the manager of the CPCRK. Informed consent was obtained from all participants, who were never coerced to participate, ensuring their right to self-determination and full disclosure. Confidentiality was upheld by protecting private information and securing all data from unauthorised access. Anonymity was maintained by using participant numbers only, preventing any link between participants and their responses.

## Results

### Demographic information

Table 1 details participants’ demographic characteristics.

**TABLE 1:** Demographic information of participants (*n* = 117).

Sociodemographic characteristics	Categories	Frequencies	
		*n*	%
Gender	Male	75	64.0
	Female	42	36.0
Age (Years)	18–24	6	5.1
	25–34	23	19.7
	35–44	28	23.9
	45–54	33	28.2
	55–64	27	23.1
Race	Black African people	48	41.3
	White people	4	3.4
	Indian people	63	54.3
	Unanswered	2	1.7
Home language	IsiZulu	42	35.9
	English	70	59.8
	Others	5	4.3
Religion	Christian	76	65.0
	Islam	15	12.8
	Hinduism	21	17.9
	Cultural	3	2.6
	Others	2	1.7
Level of education	None	2	1.7
	Primary	18	15.4
	Secondary	85	72.6
	Tertiary	12	10.3
Marital status	Single	93	79.5
	Married	19	16.2
	Divorced	2	1.7
	Widowed	2	1.7

### Adherence to treatment

To measure adherence, participants were asked if they had ever stopped their medication at any point during treatment (pre-intervention) or during the past 6 months (post-intervention). Results were compared between the pre-intervention group (*n* = 79) and the post-intervention group (*n* = 79), as well as between the post-intervention group (*n* = 79) and the control group (*n* = 30).

Pre-intervention findings showed 60.8% (*n* = 48) of the participants reported consistent adherence, while more than a third (39.2%; *n* = 31) had stopped taking their medication at some point. Post-intervention assessments revealed that 83.5% (*n* = 66) of the same participants maintained adherence over the 6 months, with only 16.5% (*n* = 13) defaulting despite the treatment buddy support. The McNemar test compared the frequency of medication stoppage at both time points and indicated a statistically significant change in adherence (*p* < 0.001), suggesting that the treatment buddy had a substantial impact on improving adherence.

A two-proportion *Z*-test showed no significant difference in treatment adherence (*p* < 0.185) between the control and post-intervention groups. Table 2 reflects the results.

**TABLE 2:** Treatment adherence comparison between pre-intervention and post-intervention and post-intervention and control groups.

Participant response	Pre-intervention (*n* = 79)		Post-intervention (*n* = 79)		Control (*n* = 30)		Differences between pre-intervention versus post-intervention groups (*p*-values)	Differences between post-intervention versus control groups (*p*-values)
	*n*	%	*n*	%	*N*	%		
Compliant (did not stop medication at any point during treatment plan)	48	60.8	66	83.5	28	93.3	< 0.001	-
Stopped their medication at some point during treatment plan	31	39.2	13	16.5	2	6.7	-	< 0.185

### Factors contributing to treatment discontinuation

All participants identified factors contributing to them defaulting on treatment, as detailed in Table 3. The total does not equate to 79, as participants could select multiple reasons.

**TABLE 3:** Factors contributing to participant’s discontinuation of psychiatric treatment (comparison between pre-intervention and post-intervention and post-intervention and control groups).

Statements	Pre-intervention (*n* = 79)		Post-intervention (*n* = 79)		Control (*n* = 30)		Differences between pre-intervention versus post-intervention (*p*-values)	Differences between post-intervention versus control (*p*-values)
	*n*	%	*n*	%	*n*	%		
Inadequate knowledge about the disease	19	24.0	2	2.5	1	3.3	< 0.001	0.819
Discouraged that the disease will not be cured (chronic nature)	15	19.3	1	1.3	1	3.3	< 0.001	0.473
Long duration of treatment	14	17.7	2	2.5	0	-	0.002	0.379
Experiencing side effects	16	20.2	2	2.5	1	3.3	< 0.001	0.819
Experiencing odd or unusual feelings, thoughts and behaviours that did not exist before illness like hearing voices, holding certain beliefs or an urge to move around	14	17.7	3	3.8	0	-	0.005	0.279
Not understanding the instructions	13	16.4	1	1.3	1	3.3	0.001	0.473
Forgetting to take the treatment	10	12.7	1	1.3	2	6.7	0.005	0.124
Suffering from substance use disorder leading to forgetting treatment for schizophrenia	5	6.3	2	2.5	1	3.3	0.246	0.819
Financial problems prevented the client from obtaining his or her medication	3	3.8	0	0.0	1	3.3	0.080	0.103
Fear of becoming labelled as worthless	2	2.5	1	1.3	1	3.3	0.560	0.473
Transport issues prevented the patient from obtaining his or her medication	2	2.5	6	7.6	1	3.3	0.147	0.418
Being unemployed led to defaulting medication	2	2.5	3	3.8	0	-	0.650	0.279
Lack of support from family	3	3.8			1	3.3	0.080	0.103
Lack of time to visit the clinic or hospital because of work or personal commitment	1	1.3	1	1.3	1	3.3	1.000	0.473
The long queue when attending the clinic or hospital caused the patient to default	1	1.3	1	1.3	0	-	1.000	0.536
The unwelcoming attitude of health care workers caused patient to default	1	1.3	0	0.0	1	3.3	0.316	0.103

The mean scores for several factors contributing to treatment discontinuation were significantly higher pre-intervention compared to post-intervention (*p* < 0.001), as shown in Table 3. This indicates a significant reduction in these factors post-intervention, including inadequate disease knowledge, discouragement regarding incurability, lengthy treatment duration, side effects, experiences of unusual sensations or thoughts, misunderstanding medication instructions and forgetfulness.

### Effect of treatment discontinuation

Table 4 displays participants’ responses on the effects of discontinuing treatment.

**TABLE 4:** Comparison of effects of discontinuing treatment between pre-intervention and post-intervention groups AND post-intervention and control groups.

Participant response	Pre-intervention number (*n* = 79)		Post-intervention (*n* = 79)		Control (*n* = 30)		Differences between pre- intervention versus post-intervention (*p*-values)	Differences between post-intervention versus control (*p*-values)
	*n*	%	*n*	%	*n*	%		
Return or worsening of symptoms of schizophrenia after partial remission	24	30.3	6	7.6	2	6.7	< 0.001	0.868
Rehospitalisation	16	20.2	3	3.8	0	0.0	0.001	0.279
Referral to specialists	11	13.9	1	1.3	0	0.0	0.003	0.536
More potent medications prescribed	11	13.9	0	0.0	0	0.0	0.001	-
Loss of employment	8	10.1	1	1.3	0	0.0	0.016	0.536
Financial loss	7	8.9	2	2.5	0	0.0	0.086	0.379
Decreased productivity in daily activities and/or chores	8	10.1	6	7.6	1	3.3	0.576	0.418
Symptoms became chronic	7	8.9	3	3.8	0	0.0	0.191	0.279
Had to stop schooling or university or studies	3	3.8	1	1.3	0	0.0	0.156	0.536
Loss of family support	4	5.0	0	0.0	0	0.0	0.043	-
Removal from the rehabilitation programme	2	2.5	0	0.0	1	3.3	0.155	0.103

Significant differences in treatment discontinuation effects were observed between pre- and post-intervention groups, showing that the negative consequences of non-adherence were substantially reduced after intervention. Participants who received the intervention reported fewer instances of symptom return or worsening, reduced rehospitalisations and specialist referrals, a lower need for more potent medication and fewer instances of job loss and diminished family support.

### Association between demographic variables and factors contributing to treatment discontinuation

In the logistic regression analysis assessing the relationship between the intervention and control groups regarding factors contributing to treatment discontinuation and its subsequent effects, no statistically significant results were found, even after adjusting for demographic factors. This indicates that, within the context of this analysis, there is insufficient evidence to assert that the intervention had a discernible impact on the adherence.

### Participants’ experience and selection of treatment buddies

Prior to intervention (*n* = 117), half of the participants (50.4%; *n* = 59) indicated a preference for assistance in adhering to treatment, while the other half felt it unnecessary. Post-intervention (*n* = 79), over two-thirds (68.3%; *n* = 54) expressed interest in using treatment buddies going forward. Regarding treatment buddy preferences, 59% (*n* = 47) favoured the psychiatric nurse who had supported them, while 41% (*n* = 32) preferred a family member or relative. In terms of decision-making for selecting a treatment buddy, 51% (*n* = 40) wanted to choose their own, 46.8% (*n* = 37) preferred a joint decision with their family, 15.8% (*n* = 12) trusted their health professional’s judgement and only 6.3% (*n* = 5) favoured a collaborative choice involving themselves, family and the health professional. Note that these percentages do not equate to 100% as some participants selected multiple options.

## Discussion

This study utilised a psychiatric nurse as a treatment buddy to remind patients to take their medication daily, dates for medication collection from the clinic and to provide support via WhatsApp or SMS. Post-intervention, adherence significantly increased, and factors contributing to previous non-adherence were reduced. Participants demonstrated improved knowledge about schizophrenia, medication control, treatment duration, side effects and intake instructions, improving their remembrance of consistent medication intake.

Additionally, the study found a significant decrease in symptom recurrence, fewer rehospitalisations, reduced specialist referrals, a lower need for more potent medications and fewer reports of loss of employment or family support. This highlights the intervention’s effectiveness in mitigating the negative effects of treatment discontinuation.

This study demonstrated that augmenting psychopharmacology with interventions supports Phan’s (2016) assertion that combining pharmacology with behavioural strategies, like patient reminders, effectively improves adherence.^36^ Thus, strategies to enhance medication use should focus on both patients and their support systems.

There were no significant differences in adherence between the control and post-intervention groups. This can be attributed to the placebo effect, as participants knew they would complete a follow-up questionnaire, and the Hawthorne effect, where the participants’ behaviour changed because of awareness of observation. Additionally, the small sample size and similar participant motivations likely influenced adherence. These factors should be considered when interpreting the study results.

Peer support programmes demonstrated improved medication adherence.^37^ Specialised psychosis programmes also resulted in better adherence than routine monthly clinic services.^38^ Additionally, treatment buddies for managing long-term HIV in Uganda and South Africa have improved adherence to antiretroviral therapy, similar to the DOT programme for tuberculosis, which became a standard method for improving adherence globally.^24,39^

Adherence in our pre-intervention group (60.7%) was higher than in other sub-Saharan low-middle-income countries like Ethiopia, Malawi and Nigeria, where adherence ranged from 45% to 59%.^9,40,41^ Treatment buddy support improved adherence (86.1%) in our study and could thus benefit patients in similar settings.

Before the intervention, nearly two-thirds (63.3%) reported inadequate knowledge of schizophrenia, contributing to non-adherence. Post-intervention, knowledge significantly improved. This is because of the treatment buddy who also provided guidance and education about the disorder, in addition to reminders of daily medication intake. Huang et al. emphasised the importance of healthcare professionals educating mental health patients about both their diagnosis and treatment.^42^ Poor disease insight is common in schizophrenia and is associated with worse outcomes in regions like Northern Ethiopia and Ghana.^43,44^ When patients fail to recognise their condition as a mental illness, they tend to be inconsistent with their treatment, which exacerbates both their symptoms and overall functioning.^45,46^ Recovery is more likely when patients acknowledge their illness and actively participate in treatment, thereby enhancing functioning within their communities.^45^

Many participants defaulted treatment because of the belief that schizophrenia is incurable. Consistent long-term treatment can improve outcomes and lead to functional remission, where symptoms decrease and social functioning improves.^47^ Within 10 years of diagnosis, half of the schizophrenia patients recover enough to live independently, while a quarter improve with support.^48^ Continuous antipsychotic maintenance is key for control, allowing patients to work, maintain relationships and function daily with minimal impairment.^49,50^ In our study, treatment buddy support significantly reduced the perception of incurability.

This study found that participants perceived schizophrenia treatment duration as too long, impacting adherence. Symptom relief often led them to stop medication, believing they were cured. Many participants defaulted within the first year, similar to findings in a Polish study where many stopped treatment within 2 to 3 months, and 80% discontinued by 2 years.^51^ This highlights the importance of early buddy support, which in our study reduced concerns about the extended treatment duration.

Participants reported that unusual feelings, like hearing voices or an urge to move, deterred them from taking medication. These residual symptoms often occur between acute episodes of schizophrenia and partial remission, with some patients continuing to hear voices or hold strange beliefs even after psychosis subsides, hindering effective management.^52,53^ Long-term low-dose antipsychotics, combined with cognitive behavioural therapy, help prevent relapse and improve mental functioning.^53^ The treatment buddy played a key role in encouraging consistent medication intake, reducing these symptoms post-intervention.

Some participants reported cognitive deficits, like forgetfulness, impacting adherence to antipsychotics. Previous studies noted that such cognitive deficits lead to partial or non-adherence, making reminders essential.^54,55,56^ Cognitive adaptation training has been suggested to improve self-care.^55^ Regular low-dose antipsychotics help maintain cognitive functioning, while high doses worsen impairment.^54^ Correct dosages are essential to prevent cognitive impairment, highlighting the value of a treatment buddy in mitigating forgetfulness and ensuring participants take their daily doses. Post-intervention, there were fewer reports of forgetfulness.

Patients experiencing antipsychotic side effects are less likely to continue treatment.^57^ This study found a significant drop in reports of side effects contributing to medication discontinuation (from 20.2% to 2.5% post-intervention), suggesting that the treatment buddy effectively helped patients understand how to manage potential side effects.

Defaulting on medication often leads to acute symptoms that require stabilisation in psychiatric emergency departments.^58^ Tareke et al. noted that non-adherence increases hospital readmissions, while Schöttle et al. found that involvement in programmes like the treatment buddy system significantly reduces involuntary admissions for schizophrenia spectrum and other psychotic disorders.^9,59^ The *South African Mental Health Care Act No. 17 of 2002* supports hospitalisation for acute symptoms, advocating for community management once stabilised.^60^ Our study found that post-intervention, reports of worsening symptoms, rehospitalisation and specialist referrals decreased, demonstrating a positive influence from the treatment buddy.

Half of the participants in our study expressed a need for assistance with medication prior to the intervention. Many individuals with mental illness in South Africa require help from family or caregivers for essential tasks like daily self-care and medication adherence.^61^ Support is crucial in schizophrenia, where strict medication adherence is required. Treatment buddies enhance adherence, helping to prevent resistance and relapse to antipsychotic drugs.

While the daily text message sent to participants was not personalised, many engaged with it, enquiring about clinic operations, logistics regarding treatment during COVID-19 and general treatment-related concerns. Others used the platform to share experiences, express appreciation and seek guidance on social issues like family violence. Some simply wanted to connect with the treatment buddy in person. Despite the intervention being primarily a reminder system, the presence of a psychiatric nurse as the virtual treatment buddy enabled appropriate responses to these interactions. This approach fostered a spontaneous patient-initiated engagement, highlighting the value of virtual support in psychiatric care.

### Limitations

Patients were recruited from a single clinic in KZN, limiting the generalisability of the results to other regions. The small control group may have impacted the outcomes; so, future studies should utilise larger and more balanced sample sizes for better power and accuracy. Notably, the primary outcome of adherence was assessed through self-reported data, which is prone to biases such as social desirability and recall bias. To mitigate these biases, anonymity and confidentiality were ensured to encourage truthful responses. Additionally, clinic nurses were interviewed to provide an external perspective on the treatment buddy programme’s influence on adherence, helping to validate self-reported data. However, the experiences reported by nurses were a separate objective of this study and are not discussed in this manuscript. Other potential biases, such as observer and response bias, may have arisen from the primary researcher’s role as the virtual treatment buddy. To safeguard against these biases, clear role separation was maintained, interactions were standardised, confidentiality was emphasised to encourage honest reporting and data collection was conducted independently to minimise the researcher’s influence.

This study employed a quasi-experimental design; hence, future research should consider true randomisation for more comparable groups and strengthened validity. A per-protocol analysis may have introduced bias by excluding non-adherent participants, affecting the generalisability of the results.

## Conclusion

Psychiatric nurses as treatment buddies are a valuable strategy for enhancing medication adherence and reducing negative outcomes in schizophrenia patients. Collaborative efforts among healthcare providers, patients and support systems are essential for effective interventions and improved overall care for individuals with schizophrenia.
